# Consequences to patients, clinicians, and manufacturers when very serious adverse drug reactions are identified (1997–2019): A qualitative analysis from the Southern Network on Adverse Reactions (SONAR)

**DOI:** 10.1016/j.eclinm.2020.100693

**Published:** 2020-12-23

**Authors:** Charles L. Bennett, Shamia Hoque, Nancy Olivieri, Matthew A. Taylor, David Aboulafia, Courtney Lubaczewski, Andrew C. Bennett, Jay Vemula, Benjamin Schooley, Bartlett J. Witherspoon, Ashley C Godwin, Paul S. Ray, Paul R. Yarnold, Henry C. Ausdenmoore, Marc Fishman, Georgne Herring, Anne Ventrone, Juan Aldaco, William J. Hrushesky, John Restaino, Henrik S. Thomsen, Paul R. Yarnold, Robert Marx, Cesar Migliorati, Salvatore Ruggiero, Chadi Nabhan, Kenneth R. Carson, June M. McKoy, Y. Tony Yang, Martin W. Schoen, Kevin Knopf, Linda Martin, Oliver Sartor, Steven Rosen, William K. Smith

**Affiliations:** a City of Hope National Medical Center in Duarte, California, United States; b University of South Carolina College of Pharmacy in Columbia, South Carolina, United States; c The SONAR Project of University of South Carolina College of Pharmacy in Columbia, South Carolina, United States; d University of South Carolina College of Engineering and Computing in Columbia, South Carolina, United States; e University of South Carolina College of Arts and Sciences in Columbia, South Carolina, United States; f University of South Carolina School of Medicine in Columbia, South Carolina, United States; g University of Miami Miller School of Medicine in Miami, Florida, United States; h Uniformed Services University F. Edward Hebert School of Medicine in Bethesda, Maryland, United States; i Tulane University School of Medicine in New Orleans, Louisiana, United States; j Northwestern University Feinberg School of Medicine in Chicago, Illinois, United States; k Rush University School of Medicine in Chicago, Illinois, United States; l Saint Louis University School of Medicine in Saint Louis, Missouri, United States; m University of Copenhagen in Copenhagen, Denmark; n Caris Life Sciences in Chicago, Illinois, United States; o Highland Hospital in Oakland, California, United States; p Virginia Mason Medical Center in Seattle, Washington, United States; q New York Center for Oral and Maxillofacial Surgery in New Hyde Park, New York, United States; r University of Florida in Gainesville, Florida, United States; s University of Toronto in Toronto, Ontario, Canada; t George Washington University School of Nursing and Milken Institute School of Public Health in Washington, District of Columbia, United States

**Keywords:** Adverse drug reaction, Liability, Patient harm, Toxicity

## Abstract

**Background:**

Adverse drug/device reactions (ADRs) can result in severe patient harm. We define very serious ADRs as being associated with severe toxicity, as measured on the Common Toxicity Criteria Adverse Events (CTCAE)) scale, following use of drugs or devices with large sales, large financial settlements, and large numbers of injured persons. We report on impacts on patients, clinicians, and manufacturers following very serious ADR reporting.

**Methods:**

We reviewed clinician identified very serious ADRs published between 1997 and 2019. Drugs and devices associated with reports of very serious ADRs were identified. Included drugs or devices had market removal discussed at Food and Drug Advisory (FDA) Advisory Committee meetings, were published by clinicians, had sales > $1 billion, were associated with CTCAE Grade 4 or 5 toxicity effects, and had either >$1 billion in settlements or >1,000 injured patients. Data sources included journals, Congressional transcripts, and news reports. We reviewed data on: 1) timing of ADR reports, Boxed warnings, and product withdrawals, and 2) patient, clinician, and manufacturer impacts. Binomial analysis was used to compare sales pre- and post-FDA Advisory Committee meetings.

**Findings:**

Twenty very serious ADRs involved fifteen drugs and one device. Legal settlements totaled $38.4 billion for 753,900 injured persons. Eleven of 18 clinicians (61%) reported harms, including verbal threats from manufacturer (five) and loss of a faculty position (one). Annual sales decreased 94% from $29.1 billion pre-FDA meeting to $4.9 billion afterwards (*p*<0.0018). Manufacturers of four drugs paid $1.7 billion total in criminal fines for failing to inform the FDA and physicians about very serious ADRs. Following FDA approval, the median time to ADR reporting was 7.5 years (Interquartile range 3,13 years). Twelve drugs received Box warnings and one drug received a warning (median, 7.5 years following ADR reporting (IQR 5,11 years). Six drugs and 1 device were withdrawn from marketing (median, 5 years after ADR reporting (IQR 4,6 years)).

**Interpretation:**

Because very serious ADRs impacts are so large, policy makers should consider developing independently funded pharmacovigilance centers of excellence to assist with clinician investigations.

**Funding:**

This work received support from the National Cancer Institute (1R01 CA102713 (CLB), https://www.nih.gov/about-nih/what-we-do/nih-almanac/national-cancer-institute-nci; and two Pilot Project grants from the American Cancer Society's Institutional Grant Award to the University of South Carolina (IRG-13–043–01) https://www.cancer.org/ (SH; BS).

**Research in context**

**Evidence before this study**

A 2001 report from the Canadian Association of University Teachers described the loss of academic professorship and settling of law suits filed by the manufacturer of deferiprone after a Canadian hematologist published reports of serious deferiprone-associated toxicity occurring in the context of a phase III manufacturer-funded clinical trial. A 2019 qualitative study evaluated consequences to patients, clinicians, and manufacturers following clinician reporting of serious cancer-related adverse drug reactions. The study, based on telephone interviews of 14 clinicians, found that 12 experienced negative feedback from manufacturers, 4 experienced negative feedback from academia, and six received either no feedback or negative feedback from the FDA.

**Added value of this study**

Nine very serious ADRs were identified during phase III clinical trials, one ADR was identified in a case-control safety study, two ADRs were identified with systematic analyses/meta-analyses, six ADRs were identified in case series developed from clinician practices; and two ADRs were identified with registries. Significant delays between clinician reporting and subsequent manufacturer/FDA notification of safety concerns were noted for 10 of 15 drugs. Thirteen safety communications were via revised product labels. United States marketing was discontinued for six drugs and one device. Over $38 billion in legal payments for drug harms were paid; 785,000 persons were purportedly injured; total annual sales decreased 94% after FDA committee hearings were held; $1.7 billion in criminal fines were paid by four manufacturers; manufacturers filed lawsuits against three clinicians; and pharmaceutical executives purportedly threatened five clinicians.

**Implications of all the available evidence**

Clinicians who publish first reports of ADRs do so at personal and professional peril. All manufacturer-funded phase III clinical trials should include truly ndependent DSMBs (without drug company representation) that have primary responsibility for ADR reporting. For clinicians who identify ADRs in practice settings, independent pharmacovigilance centers of excellence can assist with Institutional Review Board protocol applications, data analysis, communications with FDA and drug companies, with the overall goal of ameliorating the personal and professional costs to clinicians of ADR reporting.

Alt-text: Unlabelled box

## 1 Introduction

Adverse drug/device reactions (ADRs) serious enough to lead to box warnings on drug labels or drug withdrawals occur in about one fifth of all new molecular entities [1]. They can result in patient harm, affect the careers of clinicians who report these toxicities, result in substantial costs and harms to patients, and lead to large revenue losses by manufacturers. We operationally define very serious ADRs based on serious toxicity (as defined by the Common Toxicity Criteria Adverse Events scale) that follow use of drugs or devices with publicly reported $1 billion dollars in sales and/or have either a publicly reported financial safety-related payments totaling $1 billion and/or have a publicly report clinical measure of serious toxicity or death from the ADR of 1000 or more persons.

This study follows our report on implications of publishing serious hematology and oncology ADRs by clinicians [2]. Most of these ADR publications were followed by addition of Boxed warnings to product labels. Boxed warnings are the most serious warning that can be added to product labels. Careful observation by clinicians of persons who received hematology and oncology drugs and then developed unexpected syndromes led to identification of fourteen serious ADRs. As the relevant ADR had not been published previously in the literature, these clinicians were felt to be the persons most responsible for ADR identification. The study found that 83% of the fourteen clinicians reported experiencing negative feedback from manufacturers, half reported receiving negative feedback from universities or colleagues, and a third received negative feedback from regulatory officials [2].

Previously, a Canadian Association of University Teacher's report described attempts by officials at the University of Toronto to discredit the career of Nancy Olivieri, a pediatric hematologist, who published that a then under-development iron chelator, deferiprone, was associated with severe hepatotoxicity among children enrolled in a phase III clinical trial [3]. An industry-supported assessment of clinical outcomes using Dr. Olivieri's data, written by two pharmaceutical employees and one university faculty member, reported findings counter to those published by Olivieri [3]. The administration of the Hospital for Sick Children administrators removed Oliveri from her position as director of the hemoglobinopathy program there; initiated unsuceesful efforts to remove her medical license. Legal threats leading to years of litigation were settled in mediation after 18 years [3]. Adverse effects on her career have persisted for a quarter century, although her findings were confirmed by regulatory agencies including the FDA, worldwide. A 2019 retrospective study of 41 deferiprone-treated patients identified ineffectiveness and significant toxicity and deaths, in a substantial proportion of patients at a Toronto hospital [4].

We report on impacts of very serious ADRs identified between 1997 and 2019 on patients, clinicians who reported these events, and manufacturers of scrutinized drugs or devices. We extend our prior findings on hematologic and oncologic ADRs reporting to very serious ADRs. Our objective is to determine if previous accounts of physician, academic, and pharmaceutical impacts occurring after serious ADRs were published can be corroborated and better characterized.

## 2 Methods

The Southern Network on Adverse Reactions (SONAR) consists of co-investigators at fifty medical universities who have assisted with one or more evaluations of serious ADRs as part of two National Institutes of Health funded pharmacovigllance grants (1998 - 2010 and 2012 - current). Co-investigators and faculty collaborators of the co-investigators were queried about drugs or devices which satisfied the following criteria: the identified drug had large sales (generally at $100 million per year annually, but wide latitude was allowed); a clinician of whom they were aware had been the first author on the related manuscript describing a case series of a very serious ADR (operationally defined as severe organ failure or death) associated with that drug; large numbers of persons were injured as a result of the very serious ADR; and there had been some consideration that the drug might be withdrawn because of safety concerns. This search methodology formed the basis for clinicians to include in our qualitative analysis. Our main objective was to report on the personal and professional costs of publishing a manuscript describing a very serious ADR. We also evaluated events that occurred to the patients with the identified very serious ADR and to the pharmaceutical manufacturer of the implicated drug or device. The SONAR co-investigators and associated collaborators included many of the most prominent pharmacovigilance investigators in the country. Several of these individuals had testified on pharmaceutical safety before Congressional or Senate hearings on drug safety in hearings that focused on some of the drugs included in this qualitative study.

**Fig. 1:**
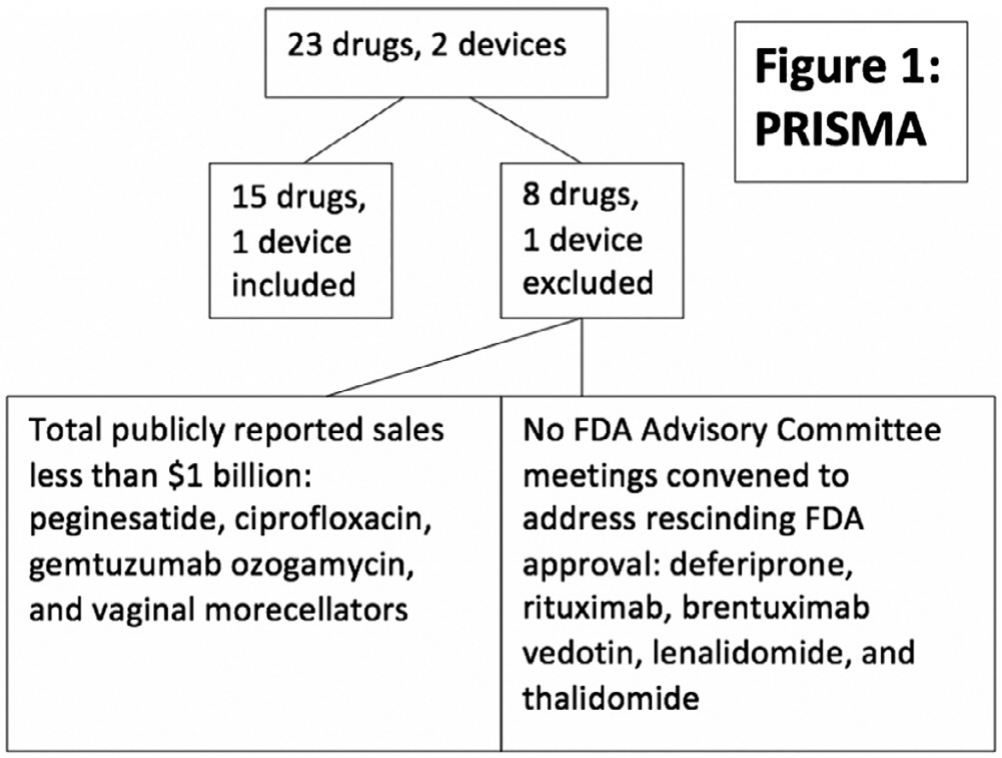
. PRISMA.

Overall, using the non-systematic approach for identification of drugs associated with very serious ADRs, SONARidentified 23 drugs and 2 devices in which rescinding of FDA marketing approval had been considered and a clinician collaborator had a primary role in uncovering these ADRs [6]. (Fig. 1) We reviewed titles and abstracts of FDA Advisory Committee meeting convened between 1997 and 2019 for meetings that focused on these drugs and devices. We searched for drugs or devices with the following characteristics: FDA Advisory Committee meeting advisors were asked to vote on recommending rescinding FDA approval for the drug or device; the initial ADR reporter was a physician who either treated persons with the relevant drug or persons who experienced the relevant toxicity and who was either the first or senior author on the ADR report; the implicated drug had publicly reported lifetime sales of $1 billion; publicly reported $1 billion in patient harm payments cited in one of the five highest US circulation newspapers (New York Times, Wall Street Journal, Chicago Tribune, Los Angeles Times, and USA Today) and had public reports of 1000 or more persons who had developed severe toxicities. Fifteen drugs and one device were included. Eight drugs and one device were excluded, although they were associated with severe ADRs. (Fig. 1) These included deferiprone, rituximab, brentuximab vedotin, lenalidomide, and thalidomide (no FDA Advisory Committee meetings were convened to address whether FDA approval should be rescinded); and peginesatide, ciprofloxacin, gemtuzumab ozogamycin, and vaginal morcellators (sales for each drug or device were less than $1 billion).

### 2.1 Definitions of very serious ADRs and study outcomes

Our definition of very serious ADRs focused on clinical, economic, and human outcomes of very serious ADR reporting. The requirement of $1 billion or more in sales is a high hurdle for pharmaceuticals, and was selected as the cut-point for sales of a single drug. The requirement of $1 billion or more in publicly reported payments was selected for a similar reason. The requirement of using a common toxicity scale, the Clinical Toxicity Criteria, and Grades 4 and 5 toxicity (Common Toxicity Criteria for Adverse Events (CTCAE). Version 5. https://ctep.cancer.gov/protocolDevelopment/electronic_applications/ctc.htm (accessed 9/4/2020) reflects serious toxicities following pharmaceutical treatments. Study co-investigators agreed that public reports of 1000 or more injured persons or fatalities represented very large human costs for drug toxicity. Sensitivity analyses found similar results if cut-points of $500 million in publicly reported total sales, $500 million in publicly reported payments to patients, and 500 or more reported persons who were severely injured. No prior study has included very serious ADRs as a topic as none of the prior literature evaluated drugs or devices that the FDA is considering rescinding regulatory approval - the most severe penalty that a manufacturer can experience related to regulatory approval decisions.

Outcomes were selected by co-investigators and included purported effects of reporting very serious ADRs on clinicians (reputational or professional harms), pharmaceutical manufacturers (loss of sales, removal of implicated drug or device from marketing, hearings with the pharmaceutical manufacturer conducted by the FDA and/or the US Congress, and criminal fines and civil settlements), and patients (publicly reported numbers of persons who were injured or who died from the very serious ADR and publicly reported financial payments related to patient injury). These outcomes were included in our study of effects of reporting serious hematologic and oncologic associated ADRs on patients, clinicians, and pharmaceutical manufacturers [2].

### 2.2 Selection of clinicians and very serious ADRs

Fifteen drugs and one device were included, as noted above. Eight drugs and one device were excluded, although they were associated with severe ADRs. (Fig. 1) These included deferiprone, rituximab, brentuximab vedotin, lenalidomide, and thalidomide (no FDA Advisory Committee meetings were convened to address whether FDA approval should be rescinded); and peginesatide, ciprofloxacin, gemtuzumab ozogamycin, and vaginal morcellators (sales for each drug or device were less than $1 billion).

We identified clinician first authors of the first and second publications describing each very serious ADR by reviewing PubMed lists of publications for the two years following the related FDA Advisory Committee meetings. As with the earlier study [2], co-investigators felt that physicians who had either treated patients with implicated drugs or devices or who treated the related toxicity were in the best position to conduct clinical ADR studies. We excluded case reports and ADR reports if reporting physicians were regulatory agency employees who did not provide patient care or if the first or last authors had not personally treated individuals with the implicated drugs/devices or individuals with the identified toxicity.

### 2.3 Data sources

Information sources on drug and device sales, number of individuals injured or who died from the very serious ADR, and payments for patient safety findings included transcripts from FDA Advisory Committees and Congressional hearings, public newspapers with circulation numbers in the top five in the United States (Top U.S. Daily Newspapers. Cision. January 4, 2019. https://web.archive.org/web/20190722203322/https://www.cision.com/us/2019/01/top-ten-us-daily-newspapers/ (accessed 8/28/2020), and corporate annual reports.

Once a drug or device associated with a very serious ADR was identified, annual reports for corporations that manufactured implicated on-patent drugs provided information on sales. Amounts of legal settlements for patient injuries were abstracted from news articles and reports in the two most highly cited scientific journals (Nature and Science)(key words “the specific drug or device” and “lawsuits/settlements”). Similar information was abstracted from transcripts of FDA Advisory Committee meetings or Congressional hearings (keywords: “identified drug,” “identified toxicity,” “identified manufacturer,” “FDA meeting,” “Congressional meeting”) and inclusion dates within two years of very serious ADR publications. These key words were used in our prior publication [2].

### 2.4 Data extraction

Three research assistants independently abstracted data for each very serious ADR.(AV, GH, HA). Weekly, research assistants and the study co-principal investigators reviewed presentations on abstracted data. Focus was placed on information on manufacturers, clinician authors’ experiences reporting ADRs, numbers of persons experiencing life-threatening or fatal toxicities, and payments for injuries. Interrater agreement was almost perfect with one disagreement adjudicated by the study co-principal investigators. PubMed listed citations for the first clinical very serious ADR reports were reviewed to identify manuscripts categorized as second reports by date. Safety-related drug or device withdrawals were identified from a publicly available index of FDA's post-market announcements and Box warning information.

We abstracted data on dates of initial FDA approval of implicated drugs or devices (obtained from Drugs@FDA and the Manufacturer and User Facility Devise Experience); publication date of initial and second very serious ADR reports; types of data sets reported in each publication; Boxed warnings, and drug or device market withdrawal. Sales data were obtained from annual reports for agents sold by publicly traded corporations (epoetin, darbepoetin, rofoecoxib, celecoxib, valdecoxib, rosiglitazone, hydroxy‑ethyol starch, and zolendroic acid) and from estimates included in newspapers (gadodiamide, hip prostheses, amd fenfluramine-phentermine). Sales data for the year prior to the first FDA Advisory Committee on safety of the implicated drug and the year following the related FDA Advisory Committee meeting (for Box warning drugs) were obtained. Post-FDA Advisory Committee meetings sales for drugs and one device with market withdrawals were set at zero, although some sales outside of the United States may have occurred. Amounts paid by manufacturers for criminal fines and civil settlements were abstracted from Department of Justice press releases.

### 2.5 Data analysis

We calculated median and ranges for time from FDA approval date to first and second very serious ADR report; safety warning; and drug withdrawal. We compared Pre/1-year versus Post-very serious ADR Report data presented in table 1 using binomial probability: the data reflect a binomial experiment with *n* = 13 independent trials, each with two possible outcomes being either success=pre>post or failure=pre<post, and with each outcome assumed to have a probability of 0.5 [6].

**Table 1:** First clinician reports of drug or device associated with very serious ADRs published one year or less before the related FDA Advisory Committee meeting or related Senate or House committee or subcommittee meeting on the ADR and funders of these studies.

Drugs with changes in product labels but not withdrawn from marketing				
sADR Reporter	# of years from FDA approval to sADR report	Drug-date withdrawn or warning added (Clinical setting that was evaluated) Group that supervised safety analyses.	Toxicity	Funder
Phase III Clinical Trial				
Leyland-Jones [13]	13 years	Epoetin –2008 (Breast cancer patients with chemotherapy-induced anemia). Independent Drug Safety Committee identified safety signal and terminated study.	Death, tumor growth among chemotherapy-treated patients who receive epoetin	Ortho Biotech
Hedenus [21]	5 years	Darbepoetin − 2008 (Lymphoid cancer patients with chemotherapy-induced anemia). Statisticians from the manufacturer analyzed study findings. No Independent Drug Safety Committee is reported in the final publication.	Overall survival decreased among chemotherapy-treated patients who receive darbepoetin	AMGEN
Leyland-Jones [13]	13 years	Epoetin – 2004 (Breast cancer patients with chemotherapy-induced anemia). Independent Drug Safety Committee identified safety signal and terminated study.	Venous thromboembolism among chemotherapy-treated patients who receive epoetin	Ortho Biotech
VanSteeneKiste [9]	2 years	Darbepoetin – 2004 (Lung cancer patients with chemotherapy induced anemia). (No independent drug safety committee identified. Co-authors included one corporate statistician, 4 corporate statisticians identified in acknowledgements, and one corporate executive (of 10 authors)).	Venous thromboembolism among chemotherapy treated patients who receive darbepoetin	AMGEN
Smith [24]	6 years	Darbepoetin – 2008 (Cancer patients with anemia without chemotherapy). Data were analysed by six co-authors (two corporate employees, two corporate consultants, one author with stock, and one with corporate research grants from the manufacturer).	Death, Cardiovascular deaths among anemic cancer patients not receiving chemotherapy	AMGEN
Singh [8]	17 years	Epoetin – 2008 (Anemic persons with diabetes and chronic kidney disease) (Corporate funded investigative team from Harvard, Duke Clinical Research Institute, and the manufacturer).	Congestive heart failure, myocardial infarction among anemic patients with chronic kidney disease	Ortho Biotech
Pfeffer [7]	7 years	Darbpoetin – 2008 (Anemic persons with diabetes and chronic kidney disease) (Corporate funded Independent Data and Safety Monitoring Committee at the University of Wisconsin)	Stroke- among anemic patients with diabetes and chronic kidney disease	AMGEN
				
				
Solomon [10]	7 years	Celecoxib – 2005 (Prevention of adenomatous polyp formation). Aware of rofecoxib's market withdrawal in Sept 2004, DSMB and Steering committee halted ACE trial and NCI established an Independent Cardiovascular Safety Committee to revew cardiovascular outcomes. In Dec 2004, NIH funded Steering Committee reported cardiovascular and mortality concerns to NCI/corporate co-funded DSMB who recommended that no more treatment with celecoxib continue. The NCI/corporate funded Steering Committee agreed to terminate treatment and to follow the study.	Death, Cardiovascular mortality	National Cancer Institute contract
META-ANALYSIS/REVIEW				
Nissen [23]	8 years	Rosiglitazone −2007 (Diabetes). Publicly available data analyzed. No IRB or Steering Committee was involved.	Death, Cardiovascular mortality	No funding
				
Case Series				
				
Ruggiero [18]	3 years	Zolendronic acid − 2004 (Skeletal metastases prevention in persons with myeloma or breast cancer). IRB Offices at Long Island Jewish and U of Maryland Hospitals approved a chart review study. No specific safety focused committee was established.	Jaw osteonecrosis	No funding
Ruggiero [18]	16 years	Pamidronate – 2004 (Skeletal metastases prevention in persons with myeloma or breast cancer). IRB Offices at Long Island Jewish and U of Maryland Hospitals approved a chart review study. No specific safety focused committee was established.	Jaw osteonecrosis	No funding
Grobner [22]	17 years	Gadodiamide 2006 (Magnetic resonance angiography). No IRB or safety committee or patients consent. Published as a case series.	Nephrogenic Systemic Fibrosis	No funding
Golomb [19]	29 years	Levofloxacin – 2015 (Serious bacterial infections of the bladder, sinus, or lung). Used an IRB approved case report form to collect data. No formal approval for the safety-focused study design.	Neuropsychiatric toxicity	No funding
				
				
Drugs or devices that were withdrawn from marketing				
Phase III Clinical Trial				
Nussmeier [12]	4 years	Valdecoxib − 2005 (Arthritis). An Independent Drug Safety Board and a Safety Committee monitored safety outcomes.	Cardiovascular toxicity	Pharmacia/ Pfizer
Perner [14]	5 years	Hydroxy-ethyl starch − 2012 (Severe sepsis patients). Had DSMB, Writing Committee, and Steering Committee. All analyses performed by one of the study authors.	Death (dialysis pts)	Danish Council
Case-control study				
Kernan [15]	62 years	Phenylpropanolamine – 2000 (Fillers included in weight loss medications and sinus medications). Three person external steering committee- none of whom were from the study team.	Hemorrhagic stroke	Roche (FDA mandated)
Meta-analysis				
Mukherjee [11]	2 years	Rofecoxib – 2004 (Arthritis). Used publicly available data. No IRB was involved.	Death, Cardiovascular mortality	No funding
Case series/registry				
Mangone [16]	13 years (registry)	Aprotonin – 2008 (Cardiac surgery patients). Steering Committee made up of study members and statisticians from the non-profit organization.	Death, Cardiovascular mortality	Non-profit organization
Connolly [17]	1 year (case series)	Fenfluramine-Phentermine – 1997 (Weight loss agent). No IRB approved study proposal. Described cases seen in practice.	Valvulopathy	No funding
Graves [20]	3 years (registry)	Prosthetic hip – 2009 . Executive committee of the Registry sends reports to regulators and manufacturers	Prosthetic failure	Australian Ministry of Health

Role of funding agencies: This study was conducted by physicians, pharmacists, nurses, epidemiologists, statisticians, attorneys, and research assistants who are affiliated with a decades-old National Institutes of Health R01 funded pharmacovigilance network called the Southern Network on Adverse Reactions (SONAR) [5]. SONAR does not accept funds from pharmaceutical manufacturers. Neither of the two funding agencies (the National Cancer Institute of Health or the American Cancer Society) had any input into the design, text, drafts, analyses, or submitted versions of the manuscript.

## 3 Results

**Fig. 2:**
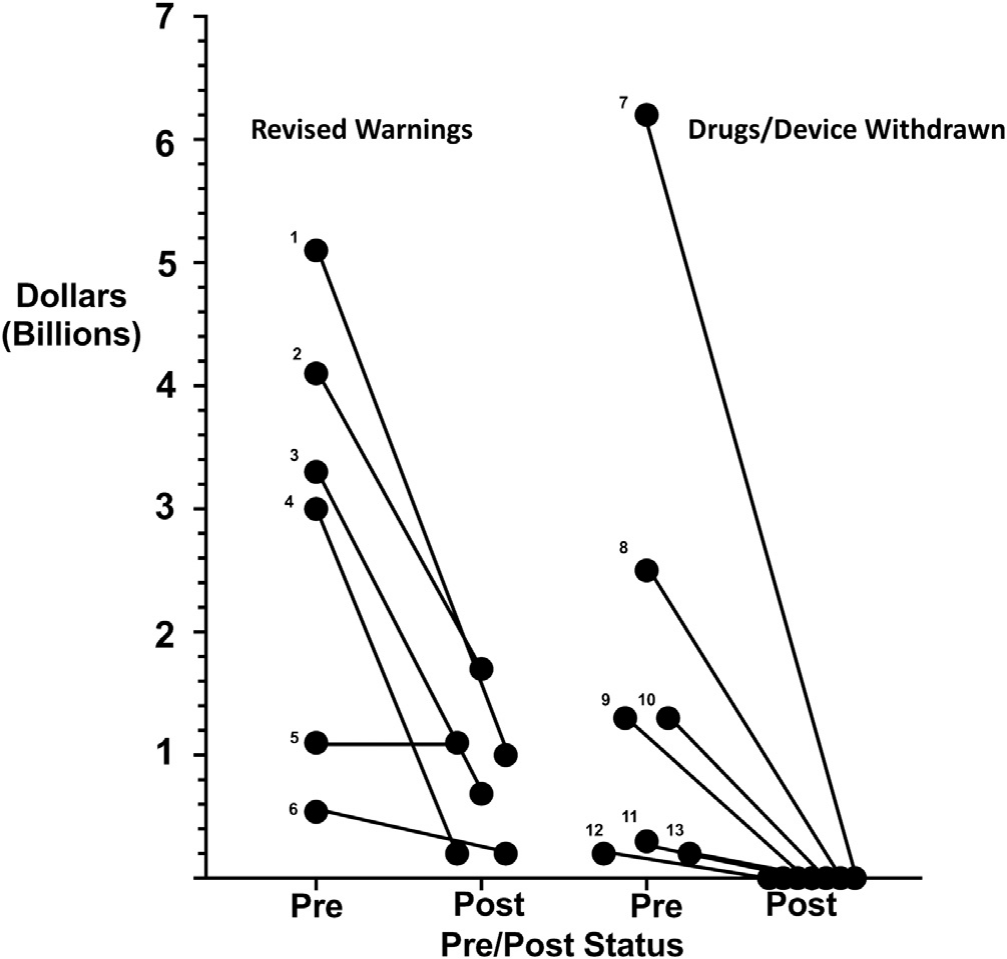
Pre/Post Revenues for Select 12 Drugs and One Device Associated with Titanic ADRs (94% Decline with *p*<0.0018).

**Fig. 3:**
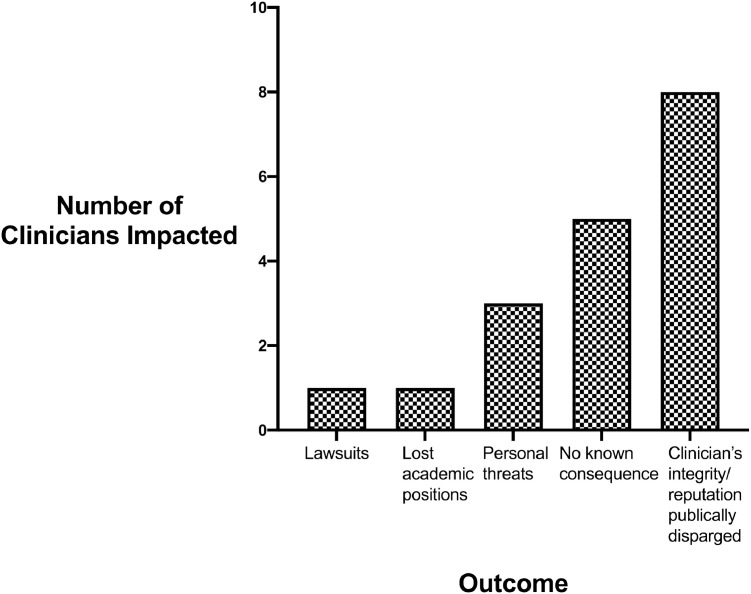
Repercussions Faced by Clinicians Reporting Titanic ADRs (*n* = 11 unique clinicians).

**Fig. 4:**
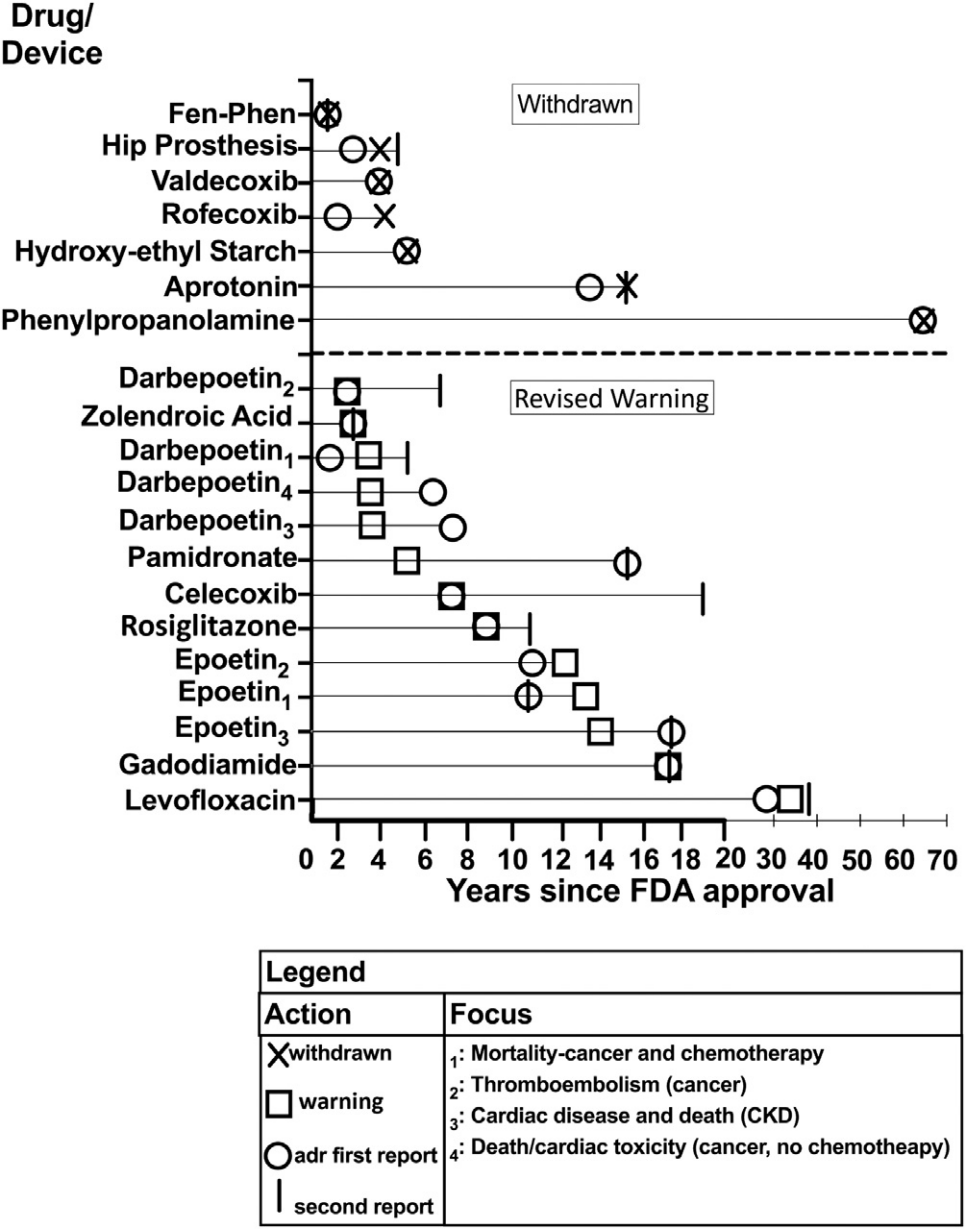
Timing of First and Second Titanic ADR Report, Box Warning, and Drug/Device Withdrawals.

Clinician reporting of very serious ADRs had significant impact on clinicians, patients, and manufacturers (Table 1, Table 2, Table 3, Fig. 2, Fig. 3, Fig. 4). Between 1997 and 2019, eighteen clinicians identified twenty very serious ADRs (Table 1, Table 2, Table 3) [7], [8], [9], [10], [11], [12], [13], [14], [15], [16], [17], [18], [19], [20], [21], [22], [23], [24]. FDA Advisory Committee hearings for very serious ADRs focused on whether marketing approval should be rescinded. All twenty very serious ADRs were discussed at one or more FDA Advisory Committee meeting. Eight very serious ADRs were discussed at two or more FDA advisory committee meetings (Table 3). Eight Congressional hearings investigated whether manufacturers purposely delayed reporting very serious ADRs. Identified toxicities included venous thromboembolism, cardiovascular events, tumor progression, jaw osteonecrosis, severe hypertension, cardiac valvulopathy, severe renal insufficiency, hemorrhagic stroke, drug-associated mortality, renal failure, severe neuropsychiatric toxicities, nephrogenic systemic fibrosis, and prosthetic hip failure. Eighteen of twenty very serious ADRs were evaluated by regulatory agencies in other countries, with concurrent agreement in almost all instances.

**Table 2:** Second published reports by clinicians of very serious ADRs.

Clinician–date of early termination (if one occurred)	DRUG	Journal-where titanic reported	Follow-up study	FDA Approval (time to ADR report)
PHASE III TRIAL				
Leyland-Jones [13]	Epoetin	Lancet-2003	Lancet-Henke-2003 [27]	1993 (10 years)
Hedenus [21]	Darbepoetin	Journal of the National Cancer Institute (JNCI)−2002	European Journal of Cancer-Overgaard-2007 [31]	2002 (1 year)
Leyland-Jones [13]	Epoetin	Lancet-2003	Blood-Vadan-Raj-2004 [35]	1993 (10 years)
VanSteenekiste [9]	Darbepoetin	JNCI-2002	Journal of Clinical Oncology-Pirker-2008- [2]	2002 (1 year)
Smith [24]	Darbepoetin	Journal of Clinical Oncology-2008	None	2002 (6 years)
Singh [8]	Epoetin	NEJM-2006	NEJM- Drueke-2006[39]	1989 (17 yrs)
Pfeffer	Darbepoetin	NEJM-2008	None	2001 (7 years)
Nussmaier [12]	Valdecoxib	NEJM-2005	None	2001 (4 years)
Perner [14]	Hydroxy-ethyl starch	NEJM-2012	None	2007 (5 years)
Solomon [10]	Celecoxib	NEJM-2005	NEJM-Nissen-2016 [30]	1998 (7 years)
CASE-CONTROL				
Kernan [15]	Phenylpropanolamine	NEJM-2000	None	1938 (62 years)
META-ANALYSIS/REVIEW				
Nissen [23]	Rosiglitazone	NEJM-2007 [40]	Lancet–Home-2009 [26]	1999 (8 years)
Mukherjee [11]	Rofecoxib	JAMA-2001 [7]	NEJM- Bresalier-2005 [40]	1999 (2 years)
OBSERVATIONAL				
Mangano [16]	Aprotinin	NEJM-2006 [19]	NEJM-Ferguson-2008 [34]	1993 (13 years)
Ruggerio [25]	Zolendronic Acid	Journal of Oral and Maxillofacial Surgery − 2004 [28]	NEJM-Durie- 2005 [38]	2001 (2 years)
Ruggerio [25]	Pamidronate	Journal of Oral and Maxillo-facial Surgery-2004 [28]	NEJM Durie-2005 [25]	1998 (15 years)
Connolly [17]	Fenfluramine-phentermine	NEJM 1997 [20]	CDC- 1997 [36]	1996 (1 year)
Grobner [22]	Gadodiamide	Nephrology Dialysis Transplation-2006 [69]	Journal of the American Society of Nephrology-Marckmann-2006 [33]	1989 (17 years)
Golomb [19]	Levafloxacin	BMJ-Open 2015 [16]	Kaur- J Clin and Supportive Oncology 2016 [37]	1986 (29 years)

Red reflects initial results that were NOT confirmed with a follow-up study.

**Table 3:** Regulatory and Congressional hearings, publicly reported costs and numbers of persons injured by the titanic ADRs.

sADR(Sales- pre/post titanic ADR report)	Clinician specialty	# of Patients	Costs	European hearing	Regulatory Hearing	Congres earings
Epoetin ($5.4 billion 2007; now $1 billion)	Hematology/oncology	NA	$761 million [58]	2007	2004,2007, 2008, 2010	2006,2011
Darbepoetin ($4.1 billion 2006/$1.7 billion 2019)	Hematology/oncology	NA	$761 million [58]	2007	2004,2007, 2008, 2010	2006,2011
Epoetin ($5.4 billion 2006 $1 billion 2019)	Hematology/oncology	NA	$761 million [58]	2007	2004,2007, 2008, 2010	2006,2011
D- + arbepoetin ($4.1 billion 2006/$1.7 billion 2019)	Hematology/oncology	NA	$761 million [58]	2007	2004,2007, 2008,2010	
Epoetin ($5.4 billion 2006/$1 billion 2019)	Nephrology	NA	$761 million [58]	2007	2004,2007,2008,2010	
Darbepoetin ($4.1 billion 2006/$1.7 billion 2019)	Nephrology	NA	$761 million [58]	2007	2004,2007, 2008, 2010	2006,2011
Valdecoxib ($1.3 billion 2003/ drug withdrawn)	Cardiovascular surgery	99,000	$785 million [57]	2004	2005	2004
Hydroxy-ethyl starch (estimated at $6.2 billion in annual sales prior to 2012) (drug withdrawn)	Intensive care	900	$560 million [59]	2017	2012	None
Celecoxib ($3.3 billion 2003/ $686 million 2018)	Surgical oncology	7000	$894 million [56]	2004	2005, 2018	2007
Phenylpropanolamine (estimated at annual sales of $200 million in 1999; drug withdrawn in 2000)	Internal medicine	NA	Not available [63]	None held	2000	None
Rosiglitazone ($3 billion 200920/$0.2 billion 2019)	Cardiology	47,000	$3400 million [50]	2010	2007, 2010, 2013	2007,2010
Rofecoxib ($2.5 billion 2003/ drug withdrawn)	Cardiology	270,000	$4850 million [51]	2004	2005	2004
Aprotonin ($0.2 billion 2006 (drug withdrawn)	Cardiology	22,000	$60 million [62]	1007, 2012	2006	None
Zolendroic acid ($1.1 billion 2004; 2020 sales estimates are $1.1 billion)	Oral surgery	Not known	Litigation pending	2005, 2009	2004	None
Pamidronate-generic (no estimated sales numbers available)	Oral surgery	Not known	Litigation pending	2005, 2009	2004	None
Fenfluramine-phentermine ($0.3 billion 1996; drug withdrawn in 1997)	Cardiology	300,000	$22,000 million [54]	None	2000	None
Gadodiamide (Sales estimates in 2006 were $0.54 billion; sales e`stimates in 2020 are $0.2 billion)	Nephrologist	Not known	Not known	2008	2009	None
Levofloxacin (generic) (estimated at greater than $1 billion while on patent; no sales estimates available for generic formulations)	Internist	NA	Not known	2018	2015-US,2018 EU	None
Articular Surface Replacement hip ($1.3 billion in 2011; withdrawn from the US in 2012)	Orthopedic surgeon	8000	$4000 million [52,53,60]	2010	2012	None

### 3.1 Data source typology

ADR findings were based on seven data source types: eight Phase III pharmaceutical funded clinical efficacy trials, one Phase III safety-focused clinical trial, one case-control study, six retrospective case series, one meta-analysis, two registry reviews, and one systematic review of published results (Table 1, Table 2, Table 3). Manuscripts describing ADRs were published a median of 7.5 years after FDA approval (Interquartile range (IQR), 3, 13 years). Peer-reviewed manuscript publications describing second reports of very serious ADRs occurred a median of 2 years following initial ADR publication (IQR 5,13 years (14 ADR reports)) [[24], [25], [26], [27],[29], [30], [31], [32], [33], [34], [35], [36], [37]] (Fig. 3). No follow-up ADR reports were published for 5 drugs voluntarily removed from the market [11,12,14,15,17]. Following FDA approval, 12 Boxed warnings and one warning describing ADRs were added a median of 7 years after FDA approval (IQR, 4,6 years) [7], [8], [9], [10], [11], [12], [13], [14], [15], [16], [17], [18], [19], [20], [21], [22], [23], [24]. In six cases, the revised warning was issued the same year as the first published report on the ADR, in four cases in the following year, in two cases one year later, in one case three years later. Manufacturers voluntarily discontinued marketing six drugs and one device associated with very serious ADRs [11,12,[14], [15], [16], [17],^20^] a median of 5 years after FDA approval (range, 1, 62 years). Four discontinuations occurred in the same year as the ADR publication, two occurred two years after publication, and one occurred three years after publication.

Clinical investigators for eight pharmaceutical-funded efficacy-focused Phase III trials, seeking label expansion for FDA-approved drugs, unexpectedly reported eight very serious ADRs involving darbepoetin, epoetin, celecoxib, rofecoxib, valdecoxib [[7], [8], [9], [10], [11], [12], [13],^21^,^24^]. Four phase III clinical trials involving epoetin, valdecoxib, and darbepoetin were terminated early for safety reasons [8,12,13,24]. One investigator of a safety-focused phase III trial for hydroxy‑ethyl starch and one investigator for an FDA mandated case-control study for phenylpropanolamine (PPA) reported ADRs [14,15]. Five clinician-reported case series described six ADRs associated with aprotonin, fenfluramine-phentermine, pamidronate, zolendroic acid, levoflxacin, and gadodiamide [[16], [17], [18], [19],^22^]. One ADR involving hip replacement devices was identified from a registry maintained by `orthopedic surgeons in Australia [20]. Studies reporting follow-up efforts to verify very serious ADR findings were published for 14 very serious ADRs at a median of two years (range, 0 to 11 years) after initial very serious ADR reports were published (Table 3, Fig. 2) [25], [26], [27], [28], [29], [30], [31], [32], [33], [34], [35], [36], [37], [38]. All but one of these studies confirmed initial very serious ADR findings. The lone exception was for rosiglitazone [23,26].

### 3.2 Clinical and economic impact of patient toxicities

Overall, 753,900 persons received payments purportedly for injuries or deaths resulting from nine very serious ADRs (Table 3). Fenfluramine-phentermine reportedly injured or resulted in death for 600,000 persons. The details of the pain, suffering and medical complications resulting from these ADRs are described in each clinical publication. For example, during the years 2000 to 2003, persons with breast cancer or multiple myeloma who developed bisphosphonate-associated osteonecrosis frequently were misdiagnosed by dentists who were not familiar with the then yet-to-be reported syndrome [18]. After months passed, patients were seen in oral surgery referral practices where debridement was undertaken. After months of treatment, some return of function and reduction in jaw pain occurred. Persons with fenfluramine-phentermine associated cardiac valvulopathy, many of whom were middle-aged women, presented with shortness of breath that worsened over several weeks to months [17]. Many affected women either died or became ill from cardiac failure. For persons with severe toxicity from epoetin or darbepoetin, presentations included difficult to treat hypertension or cardiac events in the dialysis setting or venous thromboembolism or tumor progression in the cancer setting [[7], [8], [9],^13^]. Persons with neuropsychiatric toxicity following levofloxacin administration developed agitation, difficulty concentrating, severe muscle and nerve pain, suicidal thoughts, or completed suicides [19]. Persons with gadodiamide-associated nephrogenic systemic fibrosis presented with progressive skin fibrosis and increasingly severe fibrosis of the liver, lungs, heart, and kidneys [22].

### 3.3 Clinician repercussions

Eleven of 18 clinicians (61%) reported personal or professional repercussions. Five clinicians reported receiving personal threats from executives of pharmaceutical manufacturers (for rosiglitazone, bisphosphonates, cox-2-inhibitors, and Articular Surface Replacement hips) [39], [40], [41], [42]. (Fig. 4) One Professor of Medicine lost an academic medical position after his ADR study was published [43]. Lawsuits and threats of lawsuits were reported by three clinicians [44], [45], [46]. A clinician investigator for the phenylpropanolamine-associated hemorrhagic stroke study, fearing a lawsuit, communicated findings orally to the FDA. He interpreted contract language as indicating that a lawsuit would be filed if written FDA communications preceded written manufacturer communications [44]. One clinician who reported a Phase III randomized clinical trial was sued by a pharmaceutical manufacturer for incorrectly naming the implicated product [45]. After an erratum was filed, the lawsuit was dropped. Requests by five clinicians to report their findings at FDA advisory committees were not accepted by FDA personnel who coordinated these meetings [39,41,47]. This included clinicians who had reported very serious ADRs with bisphosphonates, rosiglitazone, and aprotinin. FDA investigators articles that initial safety reports for two ADRs (associated with fenfluramine-phentermine and bisphosphonates) had been received over several months and had been included in files with many other types of adverse events for the same drugs and were difficult to interpret as causing previously unreported cardiac or oral toxicities [40,47,48].

### 3.4 Manufacturer sales losses, regulatory actions, financial payments, and efforts to discredit physicians

Annual sales for eleven drugs (epoetin, darbepoetin, valdecoxib, celecoxib, rofecoxib, rosiglitazone, aprotinin, fenfluramine-phentermine, gadodiamide, phenyl-propanolamine, zolendroic acid, gadodiamide, and hydroxy‑ethyl starch) and one device (articular surface replacements) associated with very serious ADRs decreased 94% from $29.1 billion prior to the FDA Advisory Committee meetings to $4.9 billion following these meetings (*p*<0.0018) (Table 3, Fig. 2). Following FDA Advisory Committee meetings, manufacturers voluntarily discontinued product marketing of six drugs and one device. Manufacturers paid legal settlements purportedly for patient injuries for nine drugs and one device (rosiglitazone, rofecoxib, valdecoxib, celecoxib, fenfluramine-phentermine, epoetin alfa, darbepoetin, hydroxy‑ethyl starch, gadodiamide, and ASR hips). Publicly declared lawsuit-related payments totaled $39.7 billion (Table 3) [49], [50], [51], [52], [53], [54], [55], [56], [57], [58], [59], [60], [61], [62], [63]. For fenfluramine-phentermine, $22 billion in settlements was paid to 600,000 claimants, accounting for the largest payment ever from a manufacturer for patient injury [54]. For rofecoxib-associated cardiac toxicities, one of the most prominent ADRs, the manufacturer paid $3.4 billion in settlements [51]. The manufacturer of darbepoetin and epoetin paid $615 million in legal settlements and $160 million in criminal fines, representing the largest payment ever by a biotechnology corporation [58]. For hip prostheses, the manufacturer reportedly paid $4 billion to 8000 persons for pain, suffering, and costs for hip repairs [52,53].

Manufacturer representatives publicly stated that rosiglitazone, zolendroic acic, pamidronate, epoetin, darbepoetin, articular surface replacement hips, cox-2-inhibitors, fenfluramine-phentermine, gadodiamide, and levofloxacin were unlikely causes of very serious ADRs [[39], [40], [41], [42],^44^,^47^,^48^,[64], [65], [66], [67]]. A cardiologist from the Mayo Clinic in Minnesota and a cardiologist from the Fargo Clinic in North Dakota collaboratively reported the first case series of fenfluramine-phentermine-associated valvulopathy [17]. Previously, they independently contacted the manufacturer after independently diagnosing early cases [54]. Corporate employees purportedly reported to each cardiologist that fenfluramine-phentermine was unlikely to cause cardiac valvulopathy [54]. The cardiologists’ collaborative manuscript asserted the opposite [17]. Overall, three pharmaceutical manufacturers paid $1.7 billion in criminal fines and civil settlements related in part to failure to inform the FDA and physicians about very serious ADRs associated with four drugs- valdecoxib ($1.3 billion and $1 billion, respectively), rofecoxib ($321 million and $628 million, respectively), and epoetin and darbepoetin ($150 million and $610 million, respectively) [55], [56], [57], [58].

## 4 Discussion

Very serious ADRs have significant economic, financial, and personal impacts on patients, personal and professional repercussions to physician reporters, and sales and regulatory impacts on manufacturers. In this study, the totality of the ADR impacts represented large human costs in terms of publicly reported payments for safety concerns by manufacturers, public reports of large numbers of injured persons or persons who died from ADRs, large publicly reported clinician costs in terms of loss of job or being involved in litigation with the pharmaceutical manufacturer and large decreases in product sales. While the term “very serious ADRs” is new in the medical literature, prior reports have not systematically evaluated pharmaceuticals or devices that have such large patient, clinical, and manufacturer effects. In interpreting our findings, several factors should be considered.

First, the most common reason for ADR occurrence was class-related toxicities identified for three cox-2 inhibitors, two bisphosphonates, and two erythropoiesis stimulating agents. Nine very serious ADRs were caused by six drugs (epoetin, darbepoetin, celecoxib, rofecoxib, valdecoxib, rosiglitazone) that had been evaluated in FDA Phase III licensing trials where no very serious ADRs had emerged. Subsequent Phase III trials identified nine very serious ADRs involving six drugs where study drugs were administered either at higher doses or longer durations than in Phase III licensing trials [7], [8], [9], [10], [11], [12], [13]. Fenfluramine and phentermine are two drugs that were sold to manufacturers by the French pharmaceutical company that manufactured benfluorex, a similar weight loss drug associated with tens of thousands of injured persons.

Second, our findings highlight the importance of Data Safety Monitoring Boards (DSMBs) [68]. Of 20 ADRs evaluated in this report, 40% were first reported to DSMBs evaluating relevant phase III clinical trials. DSMBs for manufacturer-funded clinical trials for valdecoxib, darbepoetin, and epoetin terminated trials early, after cardiovascular toxicity, strokes, and deaths were unexpectedly identified. Rofecoxib toxicity findings led the celecoxib study DSMB chair to pre-emptively convene a cardiovascular adjudication committee [10]. Within six weeks, medical records of patients were reviewed. Although the phase III trial was not terminated early (no safety signal was detected with the sub-study), statistically significant cardiovascular risks were identified at trial end [10]. Phase III studies of valdecoxib, with prospective assessments of cardiovascular events, were terminated by its DSMB when the manufacturer of the related drug, rofecoxib, discontinued marketing [12]. Two co-authors of this study who have been DSMB chairs noted that DSMB members are not indemnified when serving on DSMBs and protocols state that DSMB safety concerns must be reported to study sponsors who in turn report them to the FDA.[SR, OS]. In contrast, three DSMBs included corporate representatives as standing members of the DSMBs. These DSMBs reportedly delayed reporting safety events citing the observation that although deaths were greater with the study drug, investigators did not attribute these deaths to the study drug. As noted by Krumholz, Ross, and Egilman, an important lesson learned from the rofecoxib episode where the DSMB included employees of the sponsoring corporation, was that identification of serious ADRs might be delayed if non-independent DSMBs are involved [64].

Third, while corporate costs of these ADRs were large ($39.8 billion in legal fines and settlements and $24.2 billion in lost revenue), this amount represents probably less than one to two years of sales for the fifteenfifteen fifteen drugs and one device in our study. Notably, no pharmaceutical executive associated with very serious ADRs paid financial penalties for failing to disclose ADRs.

Fourth, our study identified minimal Institutional Review Board (IRB) involvement in adverse event analyses for zolendroic acid and pamidronate, levofloxacin, fenfluramide-phenteramine, and gadolinium [17,19,22,25]. Going forward, formal protocols outlining safety-focused analyses should be submitted to IRBs. Consideration should be given to forming independent drug safety centers that can assist clinicians with IRB protocol preparation and with interpreting of safety findings. These centers differ in funding, mandate, composition, and function compared with DSMBs, IRBs, FDA Advisory Committees, Steering Committees, and the FDA's Drug Oversight Board, each of whom has formal responsibilities for reviewing drug and device safety.

Our study has limitations. First, clinicians who reported very serious ADRs were not identified by name in the related warnings. While clinicians are the primary source of ADRs described in FDA databases, in 2009, FDA attributed 76% of 26 new Boxed label changes to reports from FDA employees or manufacturer employees [69]. Second, selection bias must be addressed. We did not include ADRs identified by non-clinicians or by clinicians who did not treat persons with the identified very serious ADR or who had not treated persons with implicated drugs or devices (e.g., toxicity secondary to pelvic mesh implants) as the study focused on what happens to the clinicians who identify very serious ADRs, not what happened when very serious ADRs are identified in general. Third, inclusion criteria included drugs with safety profiles reviewed by FDA advisory committees and for which the drug had annual sales of $1 billion and financial settlements of $1 billion or 1000 fatalities or injured individuals. For these ADRs, FDA Advisory Committee meetings evaluated whether FDA approvals should be rescinded. In many instances, severe side effects are identified for drugs or devices where the FDA directly requests the manufacturer add a box warning, without basing the request to revise product labels on FDA Advisory Committee's recommendations. Fourth, while each ADR manuscript had many co-authors, we evaluated only the experience of clinicians who were the first author of the first manuscript. Few news articles included interviews with authors who were not the first author of the ADR manuscript. Fifth, we reviewed print reports for five newspapers. Other more local news sources undoubtedly had additional information on each ADR. Sixth, we identified published articles disseminated within two years of pharmaceutical regulatory hearings. Finally, the identified drug and very serious ADRs were not identified by a systematic search, but were based on input from a large number of clinicians who have been collaborative with this National Institutes of Health funded pharmacovigilance initiative for over two decades. While a systematic search may have identified more drugs associated with other very serious ADRs, the current study has been conceptualized as a qualitative study that parallels in design our recent qualitative analysis of events that occurred to clinicians who reported serious hematologic and oncologyic ADRs [2]. Input from senior qualitative researchers who had published studies of “whistle-blowers” in health care recommended that between 15 and 25 clinicians would be optimal for such a qualitative study.

Our study has several strengths. Many clinicians have stated that barriers to reporting ADRs are lack of time and compensation for reporting. The implications on clinicians’ careers has rarely been described. With respect to manufacturers, medical publications generally do not report financial settlements as well as the number of patients included in these settlements. In many instances, settlements are on a one patient at a time basis and non-disclosure agreements are negotiated. Finally, review of Congressional transcripts is novel to medical studies. These transcripts provided information obtained about harms experienced by clinicians that was not available in other data sources.

Our overview supports modifications of previously proposed recommendations designed to improve ADR reporting [[69], [70], [71], [72], [73], [74], [75], [76], [77]]. First, to improve ADR reporting, clinical trials should add prospective assessments of high-likelihood ADRs (such as expected ADRs based on pre-clinical or class considerations) [76]. In some settings where safety concerns are anticipated such as for drugs in a class where serious toxicity had been identified with studies of chemically related drugs, phase III clinical trials could focus specifically on pre-identified toxicities as primary study outcomes. Second, when very serious ADRs are identified, timely safety-related responses are needed [75]. Third, clinicians should be educated that in any clinical setting, expect the unexpected toxicity when treating patients with any drug or device, as serious ADRs can first be noted at any point in the life-cycle of any drug or device [70,76]. Fourth, when causal relationships are identified, clinicians who report these findings should be shielded from personal and professional retribution [74].

This study provides answers to the related questions: “Were these very serious ADRs overlooked by the FDA during the initial drug review period?” Or “did manufacturers hide the toxicity data from the FDA and its advisors.” Our analysis suggests that both questions could be answered with a yes for ten of 15 drugs and one device in the study including epoetin, darbepoetin, rofecoxib, gadolinium, levofloxacin, rosiglitazone, zoledronic acid, pamidronate, fenfluramine-phentermine, articular surface replacement devices [77], [78], [79], [80], [81], [82], [83], [84], [85], [86], [87], [88], [89]. For four drugs (epoetin and darbepoetin, rofecoxib, and valdecoxib), three manufacturers paid $1.7 billion in criminal fines for (in part) failing to inform the FDA and physicians about serious ADRs associated with these drugs [56], [57], [58]. Details of delayed safety actions from manufacturers or the FDA have been described in Public Health Advisories, newspaper articles, transcripts from Congressional Hearings and FDA Advisory Committee meetings, and peer-reviewed scientific manuscripts. For aprotinin, the FDA issued a Public Health Advisory on September 11, 2005 indicating that the Agency was informed that the manufacturer had failed to disclose a proprietary safety database at a September 5, 2005 FDA Advisory Committee meeting [82]. For gadodiamide, attorneys discovered in 2010 that a 1993 manufacturer's report identified systemic fibrosis in gadodiamide-treated mice [83]. Manufacturers’ representatives reported that they had been notified independently by Heidi Connoly MD of the Mayo Clinic and Jack Crary MD of the Fargo Clinic of several cases of fenfluramine-phenteramine associated valvular disease in January 2006 [84]. FDA officials indicated that they had also been notified of these cases in January 2006 [84]. Representatives of the manufacturer and the FDA reported that the syndrome had never been reported to them previously and they felt that the cases did not represent an ADR [80,84]. Eric Topol MD, then of the Cleveland Clinic, reported that the manufacturer of rofecoxib had failed to communicate to the FDA cardiovascular safety concerns identified in a phase III clinical trial in 2001 [71]. Dr. Topol reported that the FDA was aware of these safety concerns in 2001, but did not respond until the drug was voluntarily withdrawn in 2004 [71]. Reporters discovered that employees of the manufacturer of rosiglitazone had written emails about its cardiovascular toxicity two years before Nissen and Wolski had described these toxicities [78]. For zolendroic acid and pamidronate, reporters wrote that three oral surgeons had submitted 93 individual adverse event reports to the FDA for two years that went unnoticed until the news article on this topic was published [18,28,38,88]. In other instances, serious ADRs were not identified until FDA approved drugs began to be evaluated at larger daily doses and/or for longer time periods. For three ADRs, bisphosphonate-associated ONJ, fenfluramine-phenteramine associated valvulopathy, and gadodiamide-associated NSF, the ADRs represented previously unreported toxicities [80,82,88].

Unhindered reporting of serious ADRs is a critical component of a high-performing healthcare system. Patients injured by the implicated drugs face severe clinical and economic hardships, many of which are permanent. However, perception of a threat to reputation or livelihood will discourage reporting of putative ADRs by clinicians. To encourage ADR reporting and protect those submitting reports, independent pharmacovigilance centers of excellence a independent drug safety board has been previously recommended by Alistair JJ Wood, Curt Furberg, and Tom Moore, three of the most prominent pharmacovigilance experts in the United States [69,73]. We recommend programs such as the Southern Network On Adverse Reports (SONAR) and its National Cancer Institute funded predecessor, the Research on Adverse Drug Events And Reports, which can assist clinicians who seek to report first cases of ADRs [5,77].

Due to the controversial nature of this work, multi-year funding for these centers of excellence with researchers and clinicians who will have multi-year appointments to the centers would protect from political pressures. Furthermore, the centers would require governmental support to provide core resources needed to faciliatate comprehensive collaborative assessments of putative ADRs.

## Declaration of Competing Interest

Dr. Sartor reports grants and personal fees from AAA, personal fees from ASTELLAS, grants and personal fees from ASTRAZENECA, grants and personal fees from BAYER, personal fees from BLUE EARTH DIAGNOSTICS, INC., personal fees from EMD SERONO, grants and personal fees from ENDOCYTE, personal fees from PFIZER, grants and personal fees from PROGENICS, grants and personal fees from SANOFI, grants from INVITAE, grants and personal fees from MERCK, grants and personal fees from NOVARTIS, grants and personal fees from JANSSEN, personal fees from CONSTELLATION, personal fees from DENDREON, personal fees from BRISTOL-MYERS SQUIBB, grants from INNOCRIN, grants from SOTIO, other from NRG, other from NCI, personal fees from BRAVARIN NORDIC, personal fees from CLOVIS, personal fees from MYRIAD, personal fees from NORIA THERAPEUTICS, INC., personal fees from NOXOPHARM, personal fees from POINT BIOPHARMA, personal fees from TENEBIO, personal fees from THERAGNOSTICS, personal fees from TELIX, personal fees from CLARITY PHARMACUEITCALS, personal fees from Celegne, personal fees from FUSION, personal fees from ISOTOPEN TECHNOLOGIEN MEUNCHEN, during the conduct of the study. All other authors report no conflict.
